# A phase 2, randomized, double‐blind, vehicle‐controlled trial of tapinarof cream in Japanese pediatric patients with atopic dermatitis

**DOI:** 10.1111/1346-8138.17587

**Published:** 2024-12-15

**Authors:** Atsuyuki Igarashi, Gaku Tsuji, Ryusei Murata, Shuichi Fukasawa, Satoshi Yamane

**Affiliations:** ^1^ Igarashi Dermatology Higashigotanda Tokyo Japan; ^2^ Research and Clinical Center for Yusho and Dioxin, Kyushu University Fukuoka Japan; ^3^ Department of Dermatology, Graduate School of Medical Sciences Kyushu University Fukuoka Japan; ^4^ Japan Tobacco Inc Tokyo Japan

**Keywords:** aryl hydrocarbon receptor (AhR), atopic dermatitis, pediatric patients, phase 2 trial, tapinarof

## Abstract

Tapinarof is a nonsteroidal, topical, aryl hydrocarbon receptor agonist approved for the treatment of atopic dermatitis (AD) in Japanese patients aged ≥12 years. We evaluated the efficacy and safety of tapinarof in Japanese pediatric patients aged 2 to 11 years with AD in a phase 2, multicenter, randomized, double‐blind, vehicle‐controlled trial. Eligible patients (*N* = 121) were randomized 1:1:1 to receive tapinarof cream 0.5%, tapinarof cream 1%, or vehicle cream once daily for 8 weeks. At week 8, the least‐squares mean percent change from baseline in Eczema Area and Severity Index (EASI) score (the primary endpoint) was −81.29% in the tapinarof 0.5% group, −77.62% in the tapinarof 1% group, and − 18.56% in the vehicle group. Reductions in EASI score at week 8 were significantly greater in the tapinarof groups than in the vehicle group (*p* < 0.0001 for both comparisons). The proportion of patients with ≥75% improvement from baseline in EASI score at week 8 was 77.5% in the tapinarof 0.5% group, 70.7% in the tapinarof 1% group, and 15.0% in the vehicle group. The proportion of patients who achieved an Investigator's Global Assessment score of 0 (clear) or 1 (almost clear) with ≥2‐grade improvement from baseline at week 8 was 32.5% in the tapinarof 0.5% group, 43.9% in the tapinarof 1% group, and 17.5% in the vehicle group. No treatment‐related serious adverse events (AEs) were reported; all of the AEs were mild or moderate. Common AEs in tapinarof‐treated patients included gastroenteritis, application site irritation, and nasopharyngitis. The incidence of trial discontinuations due to AEs was low in tapinarof‐treated patients (one patient for each strength). In summary, both strengths of tapinarof cream demonstrated greater efficacy than vehicle cream and were well tolerated in Japanese pediatric patients with AD.

## 1 INTRODUCTION

Atopic dermatitis (AD) is a common, pruritic, inflammatory skin disease characterized by recurrent eczematous lesions that substantially impairs patients' quality of life.^1^, ^2^, ^3^ AD is well known as a heterogeneous disease, the pathogenesis of which involves genetic and environmental factors, skin barrier dysfunction, and abnormal immune responses.^4^, ^5^, ^6^ In general, AD manifests in early childhood, affecting up to 25% of children and 7% to 10% of adults worldwide.^7^ In Japan, the prevalence of AD has been reported to be 5% to 27% in preschool children and 5% to 15% in elementary school children.^1^

Currently, no curative treatment of AD is available, and the primary goal of treatment is to reach and maintain stable conditions, where signs and symptoms of AD are absent or minimal without disturbance of daily activities.^1^ Topical therapies are the mainstay of treatment and are commonly used for both induction and maintenance of remission of AD. Topical therapies are generally safe but may be associated with specific adverse reactions. For example, long‐term use of topical corticosteroids can result in skin atrophy. In the past several years, nonsteroidal topical agents, such as Janus kinase inhibitors and phosphodiesterase‐4 inhibitors, have become available, and treatment options for AD have increased.^8^ Given the heterogeneity of AD, however, there remains a need for effective and safe topical agents with new mechanisms of action.

Tapinarof is a nonsteroidal, topical aryl hydrocarbon receptor (AhR) agonist.^9^ Tapinarof has been shown to activate AhR through direct binding, resulting in downregulated expression of inflammatory type 2 cytokines (e.g., interleukin [IL] 4, IL‐5, and IL‐13) and upregulated expression of skin barrier–related proteins (e.g., filaggrin, hornerin, and involucrin).^9^, ^10^ Additionally, tapinarof has been shown to activate the nuclear factor erythroid 2–related factor 2 pathway, resulting in upregulated expression of antioxidative enzymes and reduced oxidative stress.^9^, ^11^

The efficacy and safety of tapinarof cream for AD have been investigated in clinical trials. In the United States and Canada, phase 3 clinical trials of tapinarof cream have been conducted in patients aged ≥2 years with AD.^12^, ^13^ In Japan, tapinarof cream has been approved for the treatment of AD in patients aged ≥12 years.^14^ Phase 3 clinical trials in Japanese patients aged ≥12 years with AD revealed that tapinarof cream was effective and generally safe for up to 52 weeks of treatment.^15^ Tapinarof cream was also shown to potentially improve skin barrier function in patients with AD.^16^ Here, we describe the results of a phase 2 clinical trial of tapinarof cream in Japanese pediatric patients (2 to 11 years of age) with AD.

## 2 METHODS

### 2.1 Trial design

This trial was conducted in compliance with the guidelines for Good Clinical Practice and the Declaration of Helsinki. The protocol was approved by the institutional review board at each trial site. Written informed consent was provided by legal guardians of patients (and by patients, when possible).

**FIGURE 1:**
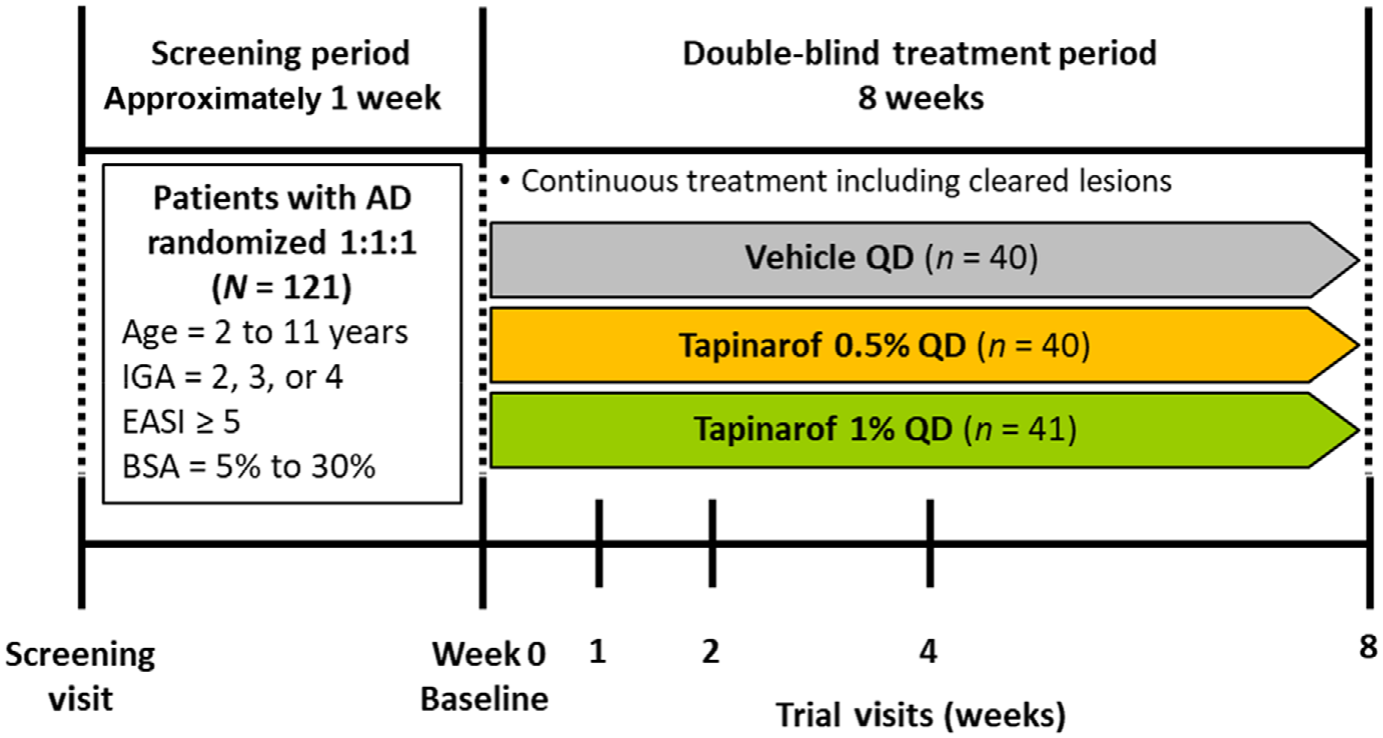
Trial design. AD, atopic dermatitis; BSA, body surface area; EASI, Eczema Area and Severity Index; IGA, Investigator's Global Assessment; QD, once daily.

This was a phase 2, multicenter, randomized, double‐blind, vehicle‐controlled trial in Japanese pediatric patients aged 2 to 11 years with AD. Patients were randomized 1:1:1 to receive tapinarof cream 0.5%, tapinarof cream 1%, or vehicle cream once daily (QD) for 8 weeks (Figure 1). A computer‐generated randomization was performed with a dynamic allocation method to balance for age category (2 to 6 years and 7 to 11 years) and Investigator's Global Assessment (IGA) score.^17^, ^18^ Patients visited trial sites at 1, 2, 4, and 8 weeks after initiation of treatment.

### 2.2 Patients

This trial enrolled Japanese patients aged 2 to 11 years with a clinical diagnosis of AD according to the criteria of the Japanese Dermatological Association.^1^ Patients were required to have an IGA score of 2 (mild), 3 (moderate), or 4 (severe); an Eczema Area and Severity Index (EASI) score^17^, ^18^, ^19^, ^20^ of ≥5 (except for the hairy scalp); and a percent of body surface area (BSA) affected between 5% and 30% (except for the hairy scalp). Patients were excluded if they had a significant dermatologic or inflammatory condition that would make it difficult to interpret data or evaluations during the trial, an acute active skin infection, or use of AD therapies within the indicated period before baseline (e.g., phototherapy, systemic corticosteroids, and immunosuppressive agents within 4 weeks, topical corticosteroids within 2 weeks or 1 week; Supporting Information Table S1).

### 2.3 Trial treatment

Legal guardians of patients (or patients, when possible) applied a thin layer of trial treatment QD to all AD lesions (except for the hairy scalp), including newly appearing lesions and lesions that improved or cleared during the trial. Therapies that were indicated for AD or that may have been effective in AD were prohibited during the trial (e.g., phototherapy, systemic corticosteroids, immunosuppressive agents, topical corticosteroids; Supporting Information Table S1). No rescue therapy was allowed for worsening of AD (therapies used for worsening of AD were regarded as a protocol deviation).

### 2.4 Trial assessments

The primary endpoint was the mean percent change from baseline in EASI score at week 8. Key secondary endpoints were the proportion of patients with ≥75% improvement from baseline in EASI score (EASI‐75) and the proportion of patients who achieved IGA treatment success, defined as an IGA score of 0 (clear) or 1 (almost clear) with ≥2‐grade improvement from baseline at week 8. In this trial, the five‐point IGA, ranging from 0 to 4, was used. Other secondary endpoints included the proportion of patients with ≥50% and ≥ 90% improvement from baseline in EASI score (EASI‐50 and EASI‐90, respectively), the proportion of patients with an IGA score of 0 or 1, the mean change from baseline in percent of BSA affected, and the mean change from baseline in daytime, nighttime, and maximum pruritus scores. The pruritus score was a five‐point scale assessment that ranged from 0 to 4,^21^ with higher scores indicating more severe itch, and was recorded by legal guardians of patients (or patients, when possible) twice daily at bedtime (daytime score) and upon awakening (nighttime score). The maximum pruritus score on an assessment day is defined as the greater of the daytime and nighttime scores.

Safety assessments included the incidence and severity of adverse events (AEs), clinical laboratory parameters, and vital signs. Concentrations of tapinarof were determined in plasma samples collected at weeks 2, 4, and 8.

### 2.5 Statistical analyses

The sample size was calculated on the basis of the results of the phase 2 trial of tapinarof in patients with AD,^22^, ^23^ where the mean percent change from baseline in EASI score at week 8 was −65.1% (standard deviation [SD], 26.1%) for tapinarof 0.5% QD, −73.6% (27.2%) for tapinarof 1% QD, and − 43.2% (41.4%) for vehicle QD. A sample size of 120 (40 per group) would provide at least 90% power for statistically significant differences (two‐sided *p* value of <0.05) between each tapinarof group and vehicle group with mixed‐effect models for repeated measures (MMRM) based on the fixed‐sequence method (in the order of the tapinarof 1% group versus the vehicle group and the tapinarof 0.5% group versus the vehicle group).

The primary analyses of efficacy were performed in the full analysis set (FAS), which consisted of all randomized patients who received at least one application of trial treatment and underwent the assessment of EASI score at least once. The primary endpoint (the mean percent change from baseline in EASI score at week 8) was analyzed with the MMRM based on the fixed‐sequence method to control the overall type I error rate at 5%. The MMRM included fixed effects for treatment group, analysis visit, treatment group by analysis visit interaction, patient as a random effect, and baseline score as a continuous covariate. An unstructured variance–covariance structure for the random effects was used for this model. The key secondary endpoints (rates of EASI‐75 response and IGA treatment success at week 8) were analyzed on the basis of the first dataset out of 100 datasets where missing data were imputed by the multiple imputation (MI) with the fully conditional specification (FCS) model. The FCS model successively imputed missing data from week 1 to 8, using linear regression models including the treatment group, baseline value, and post‐baseline values as covariates. The Fisher exact test for between‐group differences was performed. No adjustment was performed for multiple tests in the analyses of the key secondary endpoints. Other secondary endpoints were analyzed on the basis of observed cases (OC) where missing data were not imputed, and no formal statistical tests were planned for between‐group differences.

The safety analyses were performed in the safety analysis population, which consisted of all patients who received at least one application of trial treatment. Verbatim terms of AEs reported by the investigators were coded according to the Medical Dictionary for Regulatory Activities, version 24.0.

The pharmacokinetic analyses were performed in the pharmacokinetic analysis population, which consisted of all patients who had plasma concentration data of tapinarof (including plasma concentration below the lower limit of quantification [LLOQ, 50.0 pg/mL]) at ≥1 time point.

## 3 RESULTS

### 3.1 Patients

**FIGURE 2:**
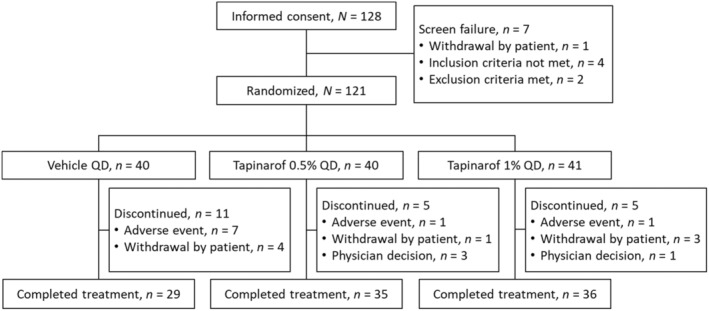
Patient disposition. QD, once daily.

A total of 121 patients were randomized to tapinarof 0.5% (*n* = 40), tapinarof 1% (*n* = 41), or vehicle (*n* = 40). All randomized patients were included in the FAS, safety analysis population, and pharmacokinetic analysis population (only patients in the tapinarof groups). More patients were prematurely discontinued from the trial in the vehicle group (*n* = 11) than in the tapinarof groups (*n* = 5 each), the primary reason for which was AEs (Figure 2). No apparent differences between treatment groups were noted in the demographics and baseline disease characteristics. At baseline, the majority of patients had an IGA score of 2 (mild) or 3 (moderate) at a similar rate; few patients had an IGA score of 4 (severe) (one or two patients in each group). The mean baseline EASI score was 11.2 (SD, 4.1) in the tapinarof 0.5% group, 11.3 (3.2) in the tapinarof 1% group, and 11.8 (4.6) in the vehicle group (Table 1).

**TABLE 1:** Patient demographics and baseline disease characteristics.

	Vehicle (*n* = 40)	Tapinarof 0.5% (*n* = 40)	Tapinarof 1% (*n* = 41)
Age, mean (SD), years	7.1 (3.1)	7.0 (2.6)	6.4 (2.8)
Age category, *n* (%)			
2–6 years	15 (37.5)	17 (42.5)	20 (48.8)
7–11 years	25 (62.5)	23 (57.5)	21 (51.2)
Male, *n* (%)	23 (57.5)	21 (52.5)	22 (53.7)
Weight, mean (SD), kg	26.3 (10.6)	24.7 (8.3)	23.1 (9.2)
BMI, mean (SD), kg/m^2^	17.2 (2.5)	16.7 (2.4)	16.5 (2.1)
Disease duration, mean (SD), years	4.4 (3.4)	3.5 (2.4)	3.4 (2.7)
EASI score, mean (SD)	11.7 (4.7)	11.2 (4.1)	11.8 (4.6)
IGA score, *n* (%)			
2: mild	19 (47.5)	19 (47.5)	20 (48.8)
3: moderate	20 (50.0)	20 (50.0)	19 (46.3)
4: severe	1 (2.5)	1 (2.5)	2 (4.9)
BSA affected, mean (SD), %	20.4 (6.7)	20.2 (6.7)	20.2 (7.2)
Pruritus score, mean (SD)			
Daytime^a^	2.0 (0.8)	2.2 (0.6)	2.4 (0.7)
Nighttime^a^	1.7 (0.8)	1.8 (0.7)	1.9 (0.5)
Maximum^b^	2.3 (0.7)	2.4 (0.5)	2.4 (0.7)

*Note*: Data based on the full analysis set.

Abbreviations: BMI, body mass index; BSA, body surface area; EASI, Eczema Area and Severity Index; IGA, Investigator's Global Assessment; SD, standard deviation.

^a^ The baseline values for daytime and nighttime pruritus scores were respectively defined as the mean values of daily daytime and nighttime pruritus scores obtained during 7 days prior to the initiation of trial treatment.

^b^ The maximum pruritus score on an assessment day is defined as the greater of the daytime and nighttime scores. The baseline value for maximum pruritus score was defined as the mean value of daily maximum pruritus scores obtained during 7 days prior to the initiation of trial treatment.

In the safety analysis population, the mean daily amount of trial treatment applied was 3.28 g (SD, 1.77 g) in the tapinarof 0.5% group, 2.96 g (1.70 g) in the tapinarof 1% group, and 3.81 g (2.62 g) in the vehicle group.

### 3.2 Efficacy

**FIGURE 3:**
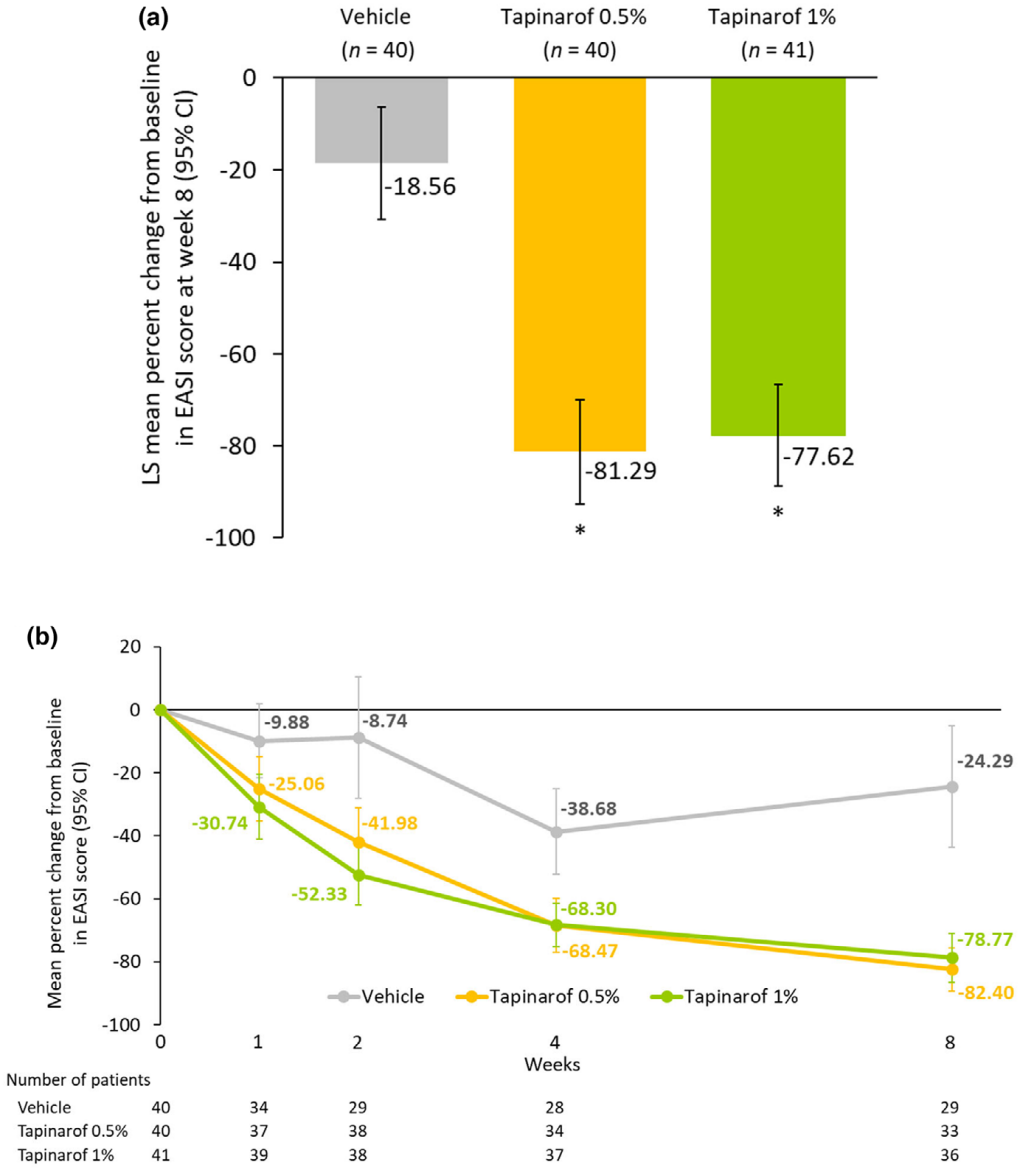
Percent change from baseline in EASI score. (a) At week 8 (the primary endpoint), analyzed with the mixed effect models for repeated measures; (b) by trial visit, analyzed on the basis of observed cases. CI, confidence interval; EASI, Eczema Area and Severity Index; LS, least‐squares. *p < 0.0001 vs vehicle.

From week 1 through week 8, greater reductions in EASI score were noted in the tapinarof groups than in the vehicle group. At week 8, the least‐squares mean percent change from baseline in EASI score was −81.29% in the tapinarof 0.5% group, −77.62% in the tapinarof 1% group, and −18.56% in the vehicle group. The difference from the vehicle group was −62.73 percent points for the tapinarof 0.5% group (95% confidence interval [CI], −79.40 to −46.05; *p* < 0.0001) and − 59.06 percent points for the tapinarof 1% group (95% CI, −75.48 to −42.64; *p* < 0.0001) (Figure 3).

**FIGURE 4:**
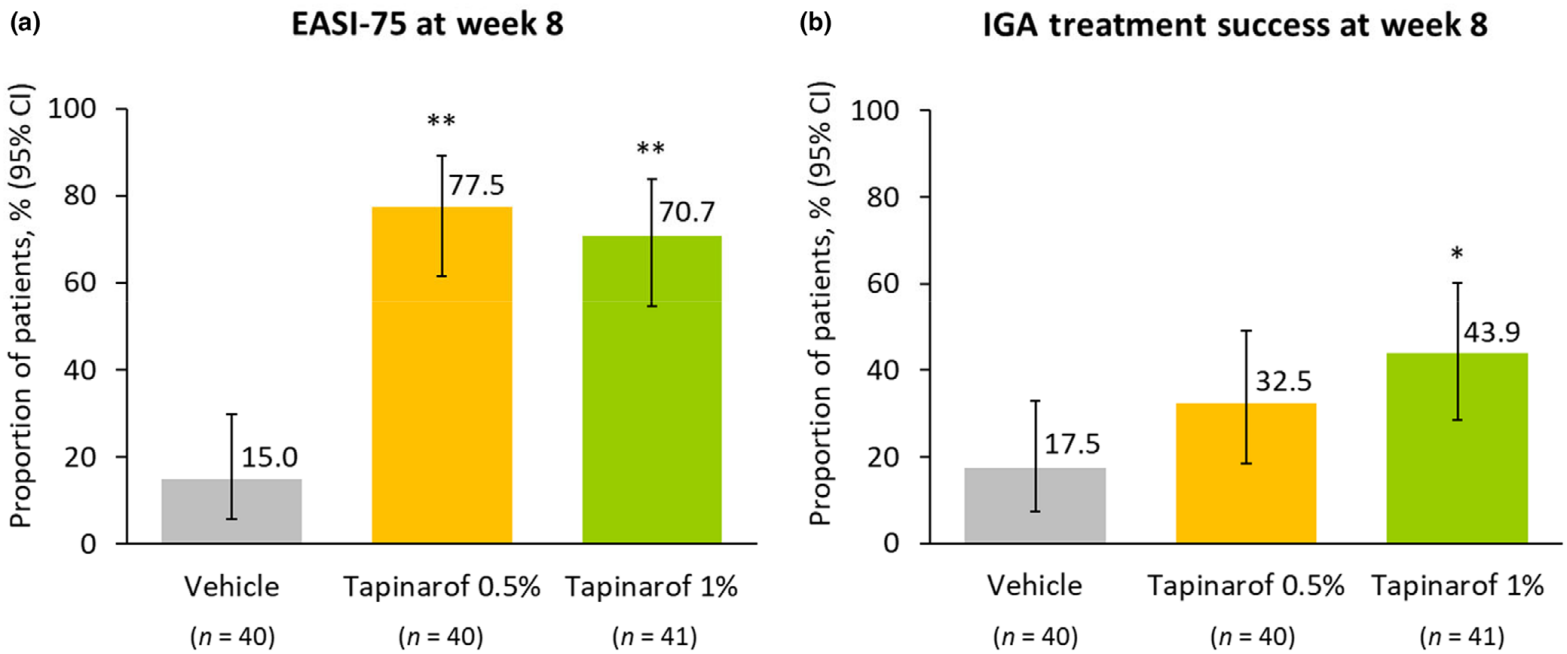
Key secondary endpoints. (a) EASI‐75 response rate at week 8, (b) IGA treatment success rate at week 8. EASI‐75 was defined as ≥75% improvement from baseline in EASI score. IGA treatment success was defined as an IGA score of 0 or 1 with ≥2‐grade improvement from baseline. Key secondary endpoints were analyzed on the basis of the first dataset out of 100 datasets where missing data were imputed by the multiple imputation. Data are presented with exact 95% CIs. *p = 0.0155 vs vehicle, **p < 0.0001 vs vehicle. CI, confidence interval; EASI, Eczema Area and Severity Index; IGA, Investigator's Global Assessment.

At week 8, EASI‐75 response rate was 77.5% in the tapinarof 0.5% group, 70.7% in the tapinarof 1% group, and 15.0% in the vehicle group. The difference from the vehicle group was 62.5 percent points for the tapinarof 0.5% group (95% CI, 40.9 to 77.9; *p* < 0.0001) and 55.7 percent points for the tapinarof 1% group (95% CI, 35.1 to 72.2; *p* < 0.0001) (Figure 4). At week 8, IGA treatment success rate was 32.5% in the tapinarof 0.5% group, 43.9% in the tapinarof 1% group, and 17.5% in the vehicle group. The difference from the vehicle group was 15.0 percent points for the tapinarof 0.5% group (95% CI, −4.5 to 34.6; *p* = 0.1961) and 26.4 percent points for the tapinarof 1% group (95% CI, 5.5 to 45.5; *p* = 0.0155) (Figure 4). Representative clinical images of patients who achieved IGA treatment success are presented in Figure S1.

**FIGURE 5:**
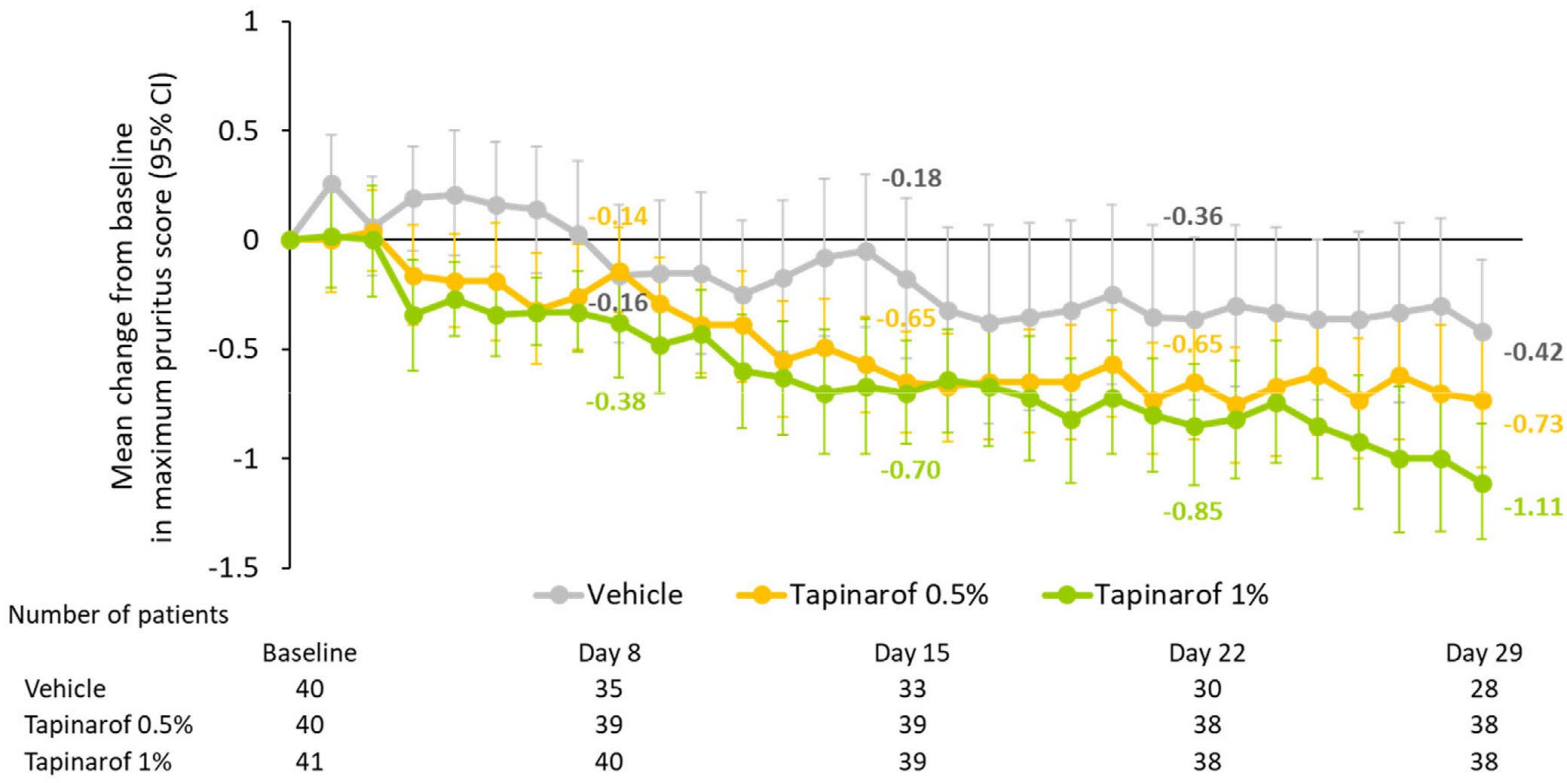
Mean daily change in maximum pruritus score from baseline to week 4. The maximum pruritus score on an assessment day is defined as the greater of the daytime and nighttime scores. The baseline value for maximum pruritus score was defined as the mean value of daily maximum pruritus scores obtained during 7 days prior to the initiation of trial treatment. CI, confidence interval.

Greater reductions in maximum pruritus score were noted shortly after initiation of treatment in the tapinarof groups, and the reductions were maintained through week 8 (Figure 5 and Supporting Information Figure S2). Improvements in other efficacy endpoints were also greater in the tapinarof groups (Supporting Information Table S2 and Figure S2). Additionally, no apparent differences in the primary and key secondary endpoints were noted between the age categories (2 to 6 years and 7 to 11 years) (Supporting Information Table S3).

### 3.3 Safety

No apparent differences between treatment groups were noted in the incidence of AEs. Overall, AEs were reported in 55.0% of patients in the tapinarof 0.5% group, 58.5% of patients in the tapinarof 1% group, and 57.5% of patients in the vehicle group (Table 2). One serious AE, asthma, was reported in the tapinarof 0.5% group; however, this event was considered unrelated to treatment. No severe AEs were reported; all AEs were mild or moderate.

**TABLE 2:** Summary of AEs.

	Vehicle (*n* = 40)	Tapinarof		
		0.5% (*n* = 40)	1% (*n* = 41)	Total (*n* = 81)
Any AEs	23 (57.5)	22 (55.0)	24 (58.5)	46 (56.8)
Serious AEs	0	1 (2.5)^a^	0	1 (1.2)
Severe AEs	0	0	0	0
Treatment‐related AEs	10 (25.0)	6 (15.0)	7 (17.1)	13 (16.0)
AEs leading to discontinuation	7 (17.5)	1 (2.5)	1 (2.4)	2 (2.5)
Most common AEs (occurring in ≥2 patients in either of the treatment group)				
Gastroenteritis	2 (5.0)	2 (5.0)	5 (12.2)	7 (8.6)
Application site irritation	5 (12.5)	2 (5.0)	3 (7.3)	5 (6.2)
Nasopharyngitis	1 (2.5)	1 (2.5)	4 (9.8)	5 (6.2)
Headache	0	1 (2.5)	3 (7.3)	4 (4.9)
Pyrexia	1 (2.5)	0	3 (7.3)	3 (3.7)
Upper respiratory tract inflammation	1 (2.5)	0	3 (7.3)	3 (3.7)
COVID‐19	2 (5.0)	2 (5.0)	0	2 (2.5)
Oral herpes	0	0	2 (4.9)	2 (2.5)
Contact dermatitis	3 (7.5)	0	1 (2.4)	1 (1.2)
Rhinitis	2 (5.0)	1 (2.5)	0	1 (1.2)
AD	6 (15.0)	0	0	0
Most common treatment‐related AEs (occurring in ≥2 patients in either of the treatment group)				
Application site irritation	5 (12.5)	2 (5.0)	3 (7.3)	5 (6.2)
Headache	0	1 (2.5)	2 (4.9)	3 (3.7)
Contact dermatitis	2 (5.0)	0	1 (2.4)	1 (1.2)
AD	4 (10.0)	0	0	0
AEs leading to discontinuation				
Application site irritation	1 (2.5)	1 (2.5)	0	1 (1.2)
Liver function test increased	0	0	1 (2.4)	1 (1.2)
AD	5 (12.5)	0	0	0
Contact dermatitis	2 (5.0)	0	0	0

*Note*: Data are presented as number of patients (percentage). The AE terms reported by the investigator were coded using Medical Dictionary for Regulatory Activities Terminology Version 24.0. All AEs are provided in Supporting Information Table S4.

Abbreviations: AD, atopic dermatitis; AE, adverse event; COVID‐19, coronavirus disease 2019.

^a^ One serious AE, asthma, was considered unrelated to treatment.

The most common AEs in the tapinarof groups included gastroenteritis (tapinarof 0.5%, *n* = 2 [5.0%]; tapinarof 1%, *n* = 5 [12.2%]), application site irritation (tapinarof 0.5%, *n* = 2 [5.0%]; tapinarof 1%, *n* = 3 [7.3%]), and nasopharyngitis (tapinarof 0.5%, *n* = 1 [2.5%]; tapinarof 1%, *n* = 4 [9.8%]), and those in the vehicle group included AD (*n* = 6 [15.0%], mostly reported as worsening of AD), application site irritation (*n* = 5 [12.5%]), and contact dermatitis (*n* = 3 [7.5%]). The most common treatment‐related AEs in the tapinarof groups included application site irritation (tapinarof 0.5%, *n* = 2 [5.0%]; tapinarof 1%, *n* = 3 [7.3%]) and headache (tapinarof 0.5%, *n* = 1 [2.5%]; tapinarof 1%, *n* = 2 [4.9%]), and those in the vehicle group included application site irritation (*n* = 5 [12.5%]) and AD (*n* = 4 [10.0%]). The incidence of trial discontinuations due to AEs was lower in the tapinarof groups (tapinarof 0.5%, *n* = 1 [2.5%]; tapinarof 1%, *n* = 1 [2.4%]) than in the vehicle group (*n* = 7 [17.5%]). The most common AE leading to trial discontinuation was AD, which was reported only in the vehicle group (*n* = 5 [12.5%]). All AEs during the trial are presented in Supporting Information Table S4.

No apparent differences in the incidence of AEs were noted between the age categories (2 to 6 years and 7 to 11 years) (Supporting Information Table S5). No clinically significant changes over time were noted in clinical laboratory parameters or vital signs.

### 3.4 Pharmacokinetics

The plasma concentration of tapinarof was below the LLOQ (50 pg/mL) in approximately 70% to 90% of patients at each time point (Supporting Information Table S6). When values below the LLOQ were treated as 0, the mean plasma concentration of tapinarof at each time point ranged from 5.14 to 349.4 pg/mL. The maximum plasma concentration of tapinarof at each time point ranged from 98.1 to 11 000 pg/mL. The patient with the highest concentration (tapinarof 0.5% group, 11 000 pg/mL at week 8) had no AEs.

## 4 DISCUSSION

In this trial, the 8‐week treatment with tapinarof cream QD led to marked improvements in the signs and symptoms of AD in a pediatric population. At week 8, the primary endpoint (the mean percent change from baseline in EASI score) and key secondary endpoints (EASI‐75 response and IGA treatment success rates) were greater for both strengths of tapinarof cream than for vehicle cream. However, IGA treatment success rate was not statistically significant for tapinarof cream 0.5%, which was likely due to a relatively small sample size for detecting a statistical difference in this efficacy endpoint. Considering the results of other efficacy endpoints together, tapinarof cream 0.5% appear to have similar efficacy to tapinarof cream 1% in treating AD in pediatric patients. Additionally, the efficacy results were consistent with those of an 8‐week treatment with tapinarof cream 1% in adolescent and adult patients (≥12 years of age) with AD.^15^

Tapinarof cream was well tolerated in pediatric patients with AD. No treatment‐related serious AEs were reported, and all of the AEs were mild or moderate. Common AEs in patients treated with tapinarof cream included gastroenteritis, application site irritation, and nasopharyngitis. The incidence of trial discontinuations due to AEs was low in patients treated with tapinarof cream (one patient for each strength). No apparent differences in the safety results were noted between the strengths of tapinarof cream. In previous trials in adolescent and adult patients (aged ≥12 years) with AD,^15^ AEs such as folliculitis, acne, and headache were frequently reported (≥10% of patients each, for up to 52 weeks of treatment), whereas the occurrences of these AEs were fewer in the present trial. Overall, tapinarof cream showed a favorable safety profile in pediatric patients with AD; nonetheless, long‐term safety results are warranted to draw conclusions on the safety of tapinarof cream in this population. A phase 3 trial of tapinarof cream for up to 60 weeks is ongoing in Japanese pediatric patients with AD (jRCT2031230139).

To our knowledge, this was the first clinical trial of tapinarof cream in Japanese pediatric patients with AD, where a relatively small number of patients were included. Further investigation in this population is required to confirm the results of the present trial. The phase 3 trial mentioned above will evaluate the efficacy and safety of tapinarof cream in an 8‐week, double‐blind, vehicle‐controlled period and also evaluate the long‐term safety and efficacy of tapinarof cream for up to 60 weeks.

In summary, in this 8‐week trial, both strengths (0.5% and 1%) of tapinarof cream demonstrated greater efficacy than vehicle cream and were well tolerated in Japanese pediatric patients with AD. No apparent differences in the efficacy and safety results were noted between the strengths of tapinarof cream; therefore, the low strength (0.5%) has been selected for further investigation in this population. The results of this trial indicate that tapinarof cream has the potential to be a promising topical treatment option for pediatric patients with AD.

## CONFLICT OF INTEREST STATEMENT

A.I. has received advisory board honoraria, consulting fees or speaker honoraria from AbbVie, Eli Lilly Japan, Japan Tobacco, Maruho, Novartis, Sanofi, LEO pharma, Otsuka Pharmaceutical, Pfizer Japan, and Torii Pharmaceutical. G.T. has received a research grant and consulting fee from Japan Tobacco. R.M., S.F., and S.Y. are employees of Japan Tobacco Inc.

## ETHICS STATEMENT

Approval of the research protocol by an institutional reviewer board: The protocol was approved by the institutional review board at each trial site. Informed consent: Written informed consent was provided by legal guardians of patients (and by patients, when possible). Animal studies: N/A.

## TRIAL REGISTRATION

The present trial is registered in Japan Registry of Clinical Trials (jRCT, https://jrct.niph.go.jp/), with the registration number of jRCT2031210257.

## Supporting information


**Data S1:** Supplementary Information.

## References

[1] Saeki H , Ohya Y , Furuta J , Arakawa H , Ichiyama S , Katsunuma T , et al. English version of clinical practice guidelines for the Management of Atopic Dermatitis 2021. J Dermatol. 2022;49:e315–e375. 35996152 10.1111/1346-8138.16527

[2] Carroll CL , Balkrishnan R , Feldman SR , Fleischer AB Jr , Manuel JC . The burden of atopic dermatitis: impact on the patient, family, and society. Pediatr Dermatol. 2005;22:192–199. 15916563 10.1111/j.1525-1470.2005.22303.x

[3] Lewis‐Jones S . Quality of life and childhood atopic dermatitis: the misery of living with childhood eczema. Int J Clin Pract. 2006;60:984–992. 16893440 10.1111/j.1742-1241.2006.01047.x

[4] Otsuka A , Nomura T , Rerknimitr P , Seidel JA , Honda T , Kabashima K . The interplay between genetic and environmental factors in the pathogenesis of atopic dermatitis. Immunol Rev. 2017;278:246–262. 28658541 10.1111/imr.12545

[5] Egawa G , Kabashima K . Multifactorial skin barrier deficiency and atopic dermatitis: essential topics to prevent the atopic march. J Allergy Clin Immunol. 2016;138:350–358. e1. 27497277 10.1016/j.jaci.2016.06.002

[6] Werfel T , Allam JP , Biedermann T , Eyerich K , Gilles S , Guttman‐Yassky E , et al. Cellular and molecular immunologic mechanisms in patients with atopic dermatitis. J Allergy Clin Immunol. 2016;138:336–349. 27497276 10.1016/j.jaci.2016.06.010

[7] Weidinger S , Beck LA , Bieber T , Kabashima K , Irvine AD . Atopic Dermatitis. Nat Rev Dis Primers. 2018;4:1. 29930242 10.1038/s41572-018-0001-z

[8] Müller S , Maintz L , Bieber T . Treatment of atopic dermatitis: recently approved drugs and advanced clinical development programs. Allergy. 2024;79:1501–1515. 38186219 10.1111/all.16009

[9] Smith SH , Jayawickreme C , Rickard DJ , Nicodeme E , Bui T , Simmons C , et al. Tapinarof is a natural AhR agonist that resolves skin inflammation in mice and humans. J Invest Dermatol. 2017;137:2110–2119. 28595996 10.1016/j.jid.2017.05.004

[10] Urashima T , Katsuda Y , Yoshiuchi H , Ebihara S , Shinozaki Y , Kato T , et al. Tapinarof, a novel topical therapeutic aryl hydrocarbon receptor agonist, suppresses atopic dermatitis‐like skin inflammation in mice. BPB Reports. 2024;7:123–131.

[11] Furue M , Hashimoto‐Hachiya A , Tsuji G . Aryl hydrocarbon receptor in atopic dermatitis and psoriasis. Int J Mol Sci. 2019;20:5424. 31683543 10.3390/ijms20215424 PMC6862295

[12] Dermavant Announces FDA Acceptance of Supplemental New Drug Application (sNDA) for VTAMA® (tapinarof) Cream, 1% for the Treatment of Atopic Dermatitis in Adults and Children 2 Years of Age and Older. [press release]. Available at: https://dermavant.com/dermavant‐announces‐fda‐acceptance‐of‐supplemental‐new‐drug‐application‐snda‐for‐vtama‐tapinarof‐cream‐1‐for‐the‐treatment‐of‐atopic‐dermatitis‐in‐adults‐and‐children‐2‐years‐of‐age‐and‐old/. Accessed 15 Oct 2024.

[13] Silverberg JI , Eichenfield LF , Hebert AA , Simpson EL , Stein Gold L , Bissonnette R , et al. Tapinarof cream 1% once daily: significant efficacy in the treatment of moderate to severe atopic dermatitis in adults and children down to 2 years of age in the pivotal phase 3 ADORING trials. J Am Acad Dermatol. 2024;91:457–465. 38777187 10.1016/j.jaad.2024.05.023

[14] JT Receives Manufacturing and Marketing Approval of VTAMA® Cream 1% for the Treatment of Atopic Dermatitis and Plaque Psoriasis in Japan. [press release]. Available at: https://www.jt.com/media/news/2024/pdf/20240624_E01.pdf. Accessed 15 Oct 2024.

[15] Igarashi A , Tsuji G , Fukasawa S , Murata R , Yamane S . Tapinarof cream for the treatment of atopic dermatitis: efficacy and safety results from two Japanese phase 3 trials. J Dermatol. 2024;51:1404–1413. 39269202 10.1111/1346-8138.17451

[16] Igarashi A , Tsuji G , Murata R , Fukasawa S , Yamane S . Improvement effects of tapinarof on the skin barrier function in Japanese patients with atopic dermatitis. J Cutan Immunol Allergy. 2024;7:13418.

[17] Rehal B , Armstrong AW . Health outcome measures in atopic dermatitis: a systematic review of trends in disease severity and quality‐of‐life instruments 1985–2010. PLoS One. 2011;6:e17520 10.1371/annotation/6d5e99c5-bd8f-4cef-b77a-fbb795633da0. Armstrong, April [Corrected to Armstrong, April W]. 21533286 PMC3076368

[18] Hill MK , Kheirandish Pishkenari A , Braunberger TL , Armstrong AW , Dunnick CA . Recent trends in disease severity and quality of life instruments for patients with atopic dermatitis: a systematic review. J Am Acad Dermatol. 2016;75:906–917. 27615798 10.1016/j.jaad.2016.07.002

[19] Hanifin JM , Thurston M , Omoto M , Cherill R , Tofte SJ , Graeber M . The eczema area and severity index (EASI): assessment of reliability in atopic dermatitis. EASI Evaluator Group Exp Dermatol. 2001;10:11–18. 11168575 10.1034/j.1600-0625.2001.100102.x

[20] Schmitt J , Spuls PI , Thomas KS , Simpson E , Furue M , Deckert S , et al. HOME initiative collaborators. The Harmonising outcome measures for eczema (HOME) statement to assess clinical signs of atopic eczema in trials. J Allergy Clin Immunol. 2014;134:800–807. 25282560 10.1016/j.jaci.2014.07.043

[21] Kawashima M , Harada S , Tango T . Evaluation of itch based on a new rating scale using patient diary. Jpn J Clin Dermatol. 2002;56:692–697. (in Japanese).

[22] Peppers J , Paller AS , Maeda‐Chubachi T , Wu S , Robbins K , Gallagher K , et al. A phase 2, randomized dose‐finding study of tapinarof (GSK2894512 cream) for the treatment of atopic dermatitis. J Am Acad Dermatol. 2019;80:89–98. e3. 30554600 10.1016/j.jaad.2018.06.047

[23] Paller AS , Stein Gold L , Soung J , Tallman AM , Rubenstein DS , Gooderham M . Efficacy and patient‐reported outcomes from a phase 2b, randomized clinical trial of tapinarof cream for the treatment of adolescents and adults with atopic dermatitis. J Am Acad Dermatol. 2021;84:632–638. 32502588 10.1016/j.jaad.2020.05.135

