# Supplementary material for: A phase 2, randomized, double‐blind, vehicle‐controlled trial of tapinarof cream in Japanese pediatric patients with atopic dermatitis
Source: J Dermatol. 2024 Dec 15;52(2):247–55. doi: 10.1111/1346-8138.17587 (PMC11807361; doi:10.1111/1346-8138.17587)
Supplement: Supplementary file 1 — Data S1: Supplementary Information. [file JDE-52-247-s001.pdf]

## SUPPORTING INFORMATION

### Supplementary Tables

**Table S1** Prohibited therapy before baseline (week 0) and during the treatment period.

| Therapy | Washout Period before Week 0 |
|---|---|
| Biologic agents that are indicated for AD or that may be effective in AD | 12 weeks or 5 half-lives (whichever is longer) |
| Unapproved drugs in Japan and other investigational products/test drugs, unapproved medical devices in Japan and other investigational devices/test devices | 12 weeks or 5 half-lives (whichever is longer) |
| Phototherapy (can be performed on the hairy scalp) | 4 weeks |
| Systemic treatments that are indicated for AD or that may be effective in AD<br>Examples: corticosteroids (including inhalant), immunosuppressive agents (e.g., cyclosporin)<br>Note: antihistamines and antiallergics (e.g., inhibitors of chemical mediator release) can be used; however, dose and dosing frequency of them should be constant from screening visit. | 4 weeks |
| Live vaccines | 4 weeks |
| Hyposensitization therapy | 4 weeks |
| Topical corticosteroids classified as strongest or very strong (can be used on the hairy scalp)[a] | 2 weeks |
| Topical treatments that are indicated for AD or that may be effective in AD (can be used on the hairy scalp)<br>Examples: topical corticosteroids classified as strong or weaker,[a] tacrolimus ointment, and delgocitinib ointment | 1 week |
| Moisturizers/protective agents (e.g., heparinoid, urea preparations, petrolatum, and zinc oxide ointment), topical antihistamines, and antiallergics at the application areas of trial treatment | from week 0 |

Abbreviation: AD, atopic dermatitis.

[a]Topical corticosteroids are classified into 5 ranks (strongest, very strong, strong, medium, and weak) according to the AD guideline in Japan.[1]

**Table S2** Summary of efficacy endpoints by trial visit.

| Treatment group | Descriptive statistics | Week 1 | Week 2 | Week 4 | Week 8 |
|---|---|---|---|---|---|
| Mean percent change from baseline in EASI score, % | | | | | |
| Vehicle (n = 40) | n | 34 | 29 | 28 | 29 |
| | Mean (SD) | −9.88 (33.78) | −8.74 (50.68) | −38.68 (35.12) | −24.29 (50.54) |
| Tapinarof 0.5% (n = 40) | n | 37 | 38 | 34 | 33 |
| | Mean (SD) | −25.06 (30.92) | −41.98 (33.47) | −68.47 (24.78) | −82.40 (19.35) |
| Tapinarof 1% (n = 41) | n | 39 | 38 | 37 | 36 |
| | Mean (SD) | −30.74 (32.13) | −52.33 (29.44) | −68.30 (20.72) | −78.77 (23.17) |
| Difference (tapinarof 0.5% vs vehicle) [95% CI] (ANOVA) | | −15.18 [−30.37, 0.01] | −33.25 [−51.71, −14.78] | −29.79 [−43.37, −16.20] | −58.11 [−74.70, −41.53] |
| Difference (tapinarof 1% vs vehicle) [95% CI] (ANOVA) | | −20.86 [−35.86, −5.86] | −43.59 [−62.06, −25.12] | −29.62 [−42.95, −16.29] | −54.49 [−70.74, −38.23] |
| EASI-50 response rate[a] | | | | | |
| Vehicle (n = 40) | n | 34 | 29 | 28 | 29 |
| | Responder, % [exact 95% CI] | 11.8 [3.3, 27.5] | 24.1 [10.3, 43.5] | 39.3 [21.5, 59.4] | 34.5 [17.9, 54.3] |
| Tapinarof 0.5% (n = 40) | n | 37 | 38 | 34 | 33 |
| | Responder, % [exact 95% CI] | 18.9 [8.0, 35.2] | 50.0 [33.4, 66.6] | 76.5 [58.8, 89.3] | 90.9 [75.7, 98.1] |
| Tapinarof 1% (n = 41) | n | 39 | 38 | 37 | 36 |
| | Responder, % [exact 95% CI] | 33.3 [19.1, 50.2] | 60.5 [43.4, 76.0] | 78.4 [61.8, 90.2] | 83.3 [67.2, 93.6] |
| Difference (tapinarof 0.5% vs vehicle) [exact 95% CI] | | 7.2 [−11.1, 25.9] | 25.9 [1.6, 47.2] | 37.2 [10.2, 59.4] | 56.4 [32.5, 74.7] |
| Difference (tapinarof 1% vs vehicle) [exact 95% CI] | | 21.6 [1.4, 40.3] | 36.4 [9.8, 56.8] | 39.1 [12.9, 60.5] | 48.9 [23.5, 68.3] |
| EASI-75 response rate[a] | | | | | |
| Vehicle (n = 40) | n | 34 | 29 | 28 | 29 |
| | Responder, % [exact 95% CI] | 0.0 [0.0, 10.3] | 3.4 [0.1, 17.8] | 21.4 [8.3, 41.0] | 20.7 [8.0, 39.7] |
| Tapinarof 0.5% (n = 40) | n | 37 | 38 | 34 | 33 |
| | Responder, % [exact 95% CI] | 2.7 [0.1, 14.2] | 13.2 [4.4, 28.1] | 44.1 [27.2, 62.1] | 78.8 [61.1, 91.0] |
| Tapinarof 1% (n = 41) | n | 39 | 38 | 37 | 36 |
| | Responder, % [exact 95% CI] | 12.8 [4.3, 27.4] | 21.1 [9.6, 37.3] | 45.9 [29.5, 63.1] | 72.2 [54.8, 85.8] |
| Difference (tapinarof 0.5% vs vehicle) [exact 95% CI] | | 2.7 [−7.9, 14.7] | 9.7 [−6.5, 25.1] | 22.7 [−2.4, 45.0] | 58.1 [32.5, 76.5] |
| Difference (tapinarof 1% vs vehicle) [exact 95% CI] | | 12.8 [1.2, 27.5] | 17.6 [0.7, 34.4] | 24.5 [−0.5, 45.8] | 51.5 [25.3, 70.6] |

| Treatment group | Descriptive statistics | Week 1 | Week 2 | Week 4 | Week 8 |
|---|---|---|---|---|---|
| EASI-90 response rate[a] | | | | | |
| Vehicle (n = 40) | n | 34 | 29 | 28 | 29 |
| | Responder, % | 0.0 | 0.0 | 7.1 | 10.3 |
| | [exact 95% CI] | [0.0, 10.3] | [0.0, 11.9] | [0.9, 23.5] | [2.2, 27.4] |
| Tapinarof 0.5% (n = 40) | n | 37 | 38 | 34 | 33 |
| | Responder, % | 0.0 | 5.3 | 23.5 | 42.4 |
| | [exact 95% CI] | [0.0, 9.5] | [0.6, 17.7] | [10.7, 41.2] | [25.5, 60.8] |
| Tapinarof 1% (n = 41) | n | 39 | 38 | 37 | 36 |
| | Responder, % | 0.0 | 5.3 | 18.9 | 44.4 |
| | [exact 95% CI] | [0.0, 9.0] | [0.6, 17.7] | [8.0, 35.2] | [27.9, 61.9] |
| Difference (tapinarof 0.5% vs vehicle) [exact 95% CI] | | NC<br>[NC, NC] | 5.3<br>[−7.1, 18.3] | 16.4<br>[−3.0, 35.0] | 32.1<br>[9.1, 52.3] |
| Difference (tapinarof 1% vs vehicle) [exact 95% CI] | | NC<br>[NC, NC] | 5.3<br>[−7.1, 18.3] | 11.8<br>[−6.8, 29.2] | 34.1<br>[9.9, 53.8] |
| IGA treatment success rate[b] | | | | | |
| Vehicle (n = 40) | n | 34 | 29 | 28 | 29 |
| | Responder, % | 0.0 | 3.4 | 7.1 | 13.8 |
| | [exact 95% CI] | [0.0, 10.3] | [0.1, 17.8] | [0.9, 23.5] | [3.9, 31.7] |
| Tapinarof 0.5% (n = 40) | n | 37 | 38 | 34 | 33 |
| | Responder, % | 0.0 | 5.3 | 20.6 | 33.3 |
| | [exact 95% CI] | [0.0, 9.5] | [0.6, 17.7] | [8.7, 37.9] | [18.0, 51.8] |
| Tapinarof 1% (n = 41) | n | 39 | 38 | 37 | 36 |
| | Responder, % | 0.0 | 2.6 | 10.8 | 41.7 |
| | [exact 95% CI] | [0.0, 9.0] | [0.1, 13.8] | [3.0, 25.4] | [25.5, 59.2] |
| Difference (tapinarof 0.5% vs vehicle) [exact 95% CI] | | NC<br>[NC, NC] | 1.8<br>[−13.1, 15.4] | 13.4<br>[−5.7, 31.7] | 19.5<br>[−2.4, 40.3] |
| Difference (tapinarof 1% vs vehicle) [exact 95% CI] | | NC<br>[NC, NC] | −0.8<br>[−15.4, 11.0] | 3.7<br>[−13.8, 19.2] | 27.9<br>[4.2, 48.4] |
| Proportion of patients with an IGA score of 0 or 1 | | | | | |
| Vehicle (n = 40) | n | 34 | 29 | 28 | 29 |
| | Responder, % | 2.9 | 3.4 | 21.4 | 31.0 |
| | [exact 95% CI] | [0.1, 15.3] | [0.1, 17.8] | [8.3, 41.0] | [15.3, 50.8] |
| Tapinarof 0.5% (n = 40) | n | 37 | 38 | 34 | 33 |
| | Responder, % | 2.7 | 10.5 | 41.2 | 63.6 |
| | [exact 95% CI] | [0.1, 14.2] | [2.9, 24.8] | [24.6, 59.3] | [45.1, 79.6] |
| Tapinarof 1% (n = 41) | n | 39 | 38 | 37 | 36 |
| | Responder, % | 2.6 | 13.2 | 35.1 | 58.3 |
| | [exact 95% CI] | [0.1, 13.5] | [4.4, 28.1] | [20.2, 52.5] | [40.8, 74.5] |
| Difference (tapinarof 0.5% vs vehicle) [exact 95% CI] | | −0.2<br>[−12.7, 12.3] | 7.1<br>[−8.7, 21.9] | 19.7<br>[−4.5, 42.3] | 32.6<br>[6.3, 54.9] |
| Difference (tapinarof 1% vs vehicle) [exact 95% CI] | | −0.4<br>[−12.9, 10.9] | 9.7<br>[−6.5, 25.1] | 13.7<br>[−9.4, 35.2] | 27.3<br>[1.6, 49.4] |

| Treatment group | Descriptive statistics | Week 1 | Week 2 | Week 4 | Week 8 |
|---|---|---|---|---|---|
| Mean change from baseline in %BSA affected | | | | | |
| Vehicle | n | 34 | 29 | 28 | 29 |
| (n = 40) | Mean (SD) | −1.2 (5.4) | −1.0 (11.2) | −5.0 (9.0) | −5.4 (10.1) |
| Tapinarof 0.5% | n | 37 | 38 | 34 | 33 |
| (n = 40) | Mean (SD) | −1.9 (5.9) | −5.5 (7.2) | −10.4 (8.3) | −14.1 (7.9) |
| Tapinarof 1% | n | 39 | 38 | 37 | 36 |
| (n = 41) | Mean (SD) | −3.2 (5.6) | −7.4 (6.5) | −10.5 (6.5) | −12.8 (6.7) |
| Difference (tapinarof 0.5% vs vehicle) [95% CI] (ANOVA) | | −0.7 [−3.4, 1.9] | −4.6 [−8.6, −0.5] | −5.3 [−9.3, −1.3] | −8.7 [−12.9, −4.5] |
| Difference (tapinarof 1% vs vehicle) [95% CI] (ANOVA) | | −2.0 [−4.6, 0.6] | −6.5 [−10.5, −2.4] | −5.5 [−9.4, −1.6] | −7.4 [−11.4, −3.3] |
| Mean change from baseline in daytime pruritus score[c] | | | | | |
| Vehicle | n | 35 | 29 | 28 | 29 |
| (n = 40) | Mean (SD) | 0.30 (0.73) | 0.14 (0.88) | −0.24 (0.91) | −0.09 (0.95) |
| Tapinarof 0.5% | n | 38 | 38 | 34 | 33 |
| (n = 40) | Mean (SD) | −0.11 (0.54) | −0.30 (0.62) | −0.56 (0.86) | −1.03 (0.95) |
| Tapinarof 1% | n | 39 | 38 | 37 | 36 |
| (n = 41) | Mean (SD) | −0.21 (0.52) | −0.62 (0.70) | −0.86 (0.81) | −1.23 (0.85) |
| Difference (tapinarof 0.5% vs vehicle) [95% CI] (ANOVA) | | −0.41 [−0.69, −0.13] | −0.44 [−0.80, −0.08] | −0.32 [−0.75, 0.11] | −0.94 [−1.40, −0.47] |
| Difference (tapinarof 1% vs vehicle) [95% CI] (ANOVA) | | −0.51 [−0.79, −0.23] | −0.76 [−1.12, −0.41] | −0.61 [−1.04, −0.19] | −1.13 [−1.59, −0.68] |
| Mean change from baseline in nighttime pruritus score[c] | | | | | |
| Vehicle | n | 35 | 29 | 28 | 29 |
| (n = 40) | Mean (SD) | 0.08 (0.68) | −0.18 (0.99) | −0.34 (0.85) | −0.39 (0.85) |
| Tapinarof 0.5% | n | 38 | 38 | 34 | 33 |
| (n = 40) | Mean (SD) | −0.18 (0.50) | −0.36 (0.49) | −0.56 (0.72) | −0.92 (0.85) |
| Tapinarof 1% | n | 39 | 38 | 37 | 36 |
| (n = 41) | Mean (SD) | −0.15 (0.59) | −0.36 (0.60) | −0.59 (0.79) | −0.90 (0.75) |
| Difference (tapinarof 0.5% vs vehicle) [95% CI] (ANOVA) | | −0.26 [−0.54, 0.01] | −0.18 [−0.52, 0.16] | −0.22 [−0.62, 0.17] | −0.53 [−0.94, −0.12] |
| Difference (tapinarof 1% vs vehicle) [95% CI] (ANOVA) | | −0.23 [−0.50, 0.04] | −0.18 [−0.52, 0.16] | −0.25 [−0.64, 0.14] | −0.51 [−0.91, −0.10] |

| Treatment group | Descriptive statistics | Week 1 | Week 2 | Week 4 | Week 8 |
|---|---|---|---|---|---|
| Mean change from baseline in maximum pruritus score[d] | | | | | |
| Vehicle | n | 35 | 29 | 28 | 29 |
| (n = 40) | Mean (SD) | 0.17 (0.69) | −0.06 (0.94) | −0.44 (0.92) | −0.29 (0.83) |
| Tapinarof 0.5% | n | 38 | 38 | 34 | 33 |
| (n = 40) | Mean (SD) | −0.17 (0.56) | −0.39 (0.58) | −0.65 (0.76) | −1.14 (0.92) |
| Tapinarof 1% | n | 39 | 38 | 37 | 36 |
| (n = 41) | Mean (SD) | −0.22 (0.45) | −0.61 (0.63) | −0.87 (0.77) | −1.23 (0.83) |
| Difference (tapinarof 0.5% vs vehicle) [95% CI] (ANOVA) | | −0.34 [−0.60, −0.07] | −0.32 [−0.67, 0.02] | −0.21 [−0.62, 0.20] | −0.85 [−1.28, −0.41] |
| Difference (tapinarof 1% vs vehicle) [95% CI] (ANOVA) | | −0.39 [−0.66, −0.13] | −0.54 [−0.89, −0.19] | −0.43 [−0.83, −0.02] | −0.94 [−1.37, −0.52] |

Abbreviations: ANOVA, analysis of variance; BSA, body surface area; CI, confidence interval; EASI, Eczema Area and Severity Index; IGA, Investigator's Global Assessment; NC, not calculated; SD, standard deviation.

All efficacy endpoints by study visit were analyzed on the basis of observed cases where missing data were not imputed.

[a]EASI-50, -75, and -90 were defined as ≥ 50%, ≥ 75%, and ≥ 90% improvement from baseline in EASI score, respectively.

[b]IGA treatment success was defined as an IGA score of 0 or 1 with ≥ 2-grade improvement from baseline.

[c]The baseline values for daytime and nighttime pruritus scores were respectively defined as the mean values of daily daytime and nighttime pruritus scores obtained during 7 days prior to the initiation of trial treatment. The daytime and nighttime pruritus scores at each visit were respectively defined as the mean values of daily daytime and nighttime pruritus scores obtained during 7 days prior to the visit.

[d]The maximum pruritus score on an assessment day is defined as the greater of the daytime and nighttime scores. The baseline value for maximum pruritus score was defined as the mean value of daily maximum pruritus scores obtained during 7 days prior to the initiation of trial treatment. The maximum pruritus score at each visit was defined as the mean value of daily maximum pruritus scores obtained during 7 days prior to the relevant visit.

**Table S3** Summary of efficacy endpoints at week 8 by age category.

| Age category | Descriptive statistics | Vehicle (n = 40) | Tapinarof 0.5% (n = 40) | Tapinarof 1% (n = 41) |
|---|---|---|---|---|
| Mean percent change from baseline in EASI score, % | | | | |
| | n | 9 | 15 | 17 |
| 2-6 years | Mean (SD) | −19.05 (65.50) | −81.25 (21.60) | −79.66 (21.17) |
| | [95% CI] | [−69.40, 31.30] | [−93.21, −69.29] | [−90.54, −68.77] |
| | n | 20 | 18 | 19 |
| 7-11 years | Mean (SD) | −26.64 (44.04) | −83.36 (17.86) | −77.99 (25.38) |
| | [95% CI] | [−47.25, −6.03] | [−92.24, −74.48] | [−90.22, −65.75] |
| EASI-75 response rate[a] | | | | |
| | n | 9 | 15 | 17 |
| 2-6 years | Responder, % | 33.3 | 73.3 | 70.6 |
| | [exact 95% CI] | [7.5, 70.1] | [44.9, 92.2] | [44.0, 89.7] |
| | n | 20 | 18 | 19 |
| 7-11 years | Responder, % | 15.0 | 83.3 | 73.7 |
| | [exact 95% CI] | [3.2, 37.9] | [58.6, 96.4] | [48.8, 90.9] |
| IGA treatment success rate[b] | | | | |
| | n | 9 | 15 | 17 |
| 2-6 years | Responder, % | 22.2 | 26.7 | 29.4 |
| | [exact 95% CI] | [2.8, 60.0] | [7.8, 55.1] | [10.3, 56.0] |
| | n | 20 | 18 | 19 |
| 7-11 years | Responder, % | 10.0 | 38.9 | 52.6 |
| | [exact 95% CI] | [1.2, 31.7] | [17.3, 64.3] | [28.9, 75.6] |

Abbreviations: CI, confidence interval; EASI, Eczema Area and Severity Index; IGA, Investigator's Global Assessment; SD, standard deviation.

All efficacy endpoints were analyzed on the basis of observed cases where missing data were not imputed.

[a]EASI-75 was defined as ≥ 75% improvement from baseline in EASI score.

[b]IGA treatment success was defined as an IGA score of 0 or 1 with ≥ 2-grade improvement from baseline.

**Table S4** All adverse events.

| AE terms | Vehicle (n = 40) | Tapinarof | | |
|---|---|---|---|---|
| | | **0.5% (n = 40)** | **1% (n = 41)** | **Total (n = 81)** |
| Gastroenteritis | 2 (5.0) | 2 (5.0) | 5 (12.2) | 7 (8.6) |
| Application site irritation | 5 (12.5) | 2 (5.0) | 3 (7.3) | 5 (6.2) |
| Nasopharyngitis | 1 (2.5) | 1 (2.5) | 4 (9.8) | 5 (6.2) |
| Headache | 0 | 1 (2.5) | 3 (7.3) | 4 (4.9) |
| Pyrexia | 1 (2.5) | 0 | 3 (7.3) | 3 (3.7) |
| Upper respiratory tract inflammation | 1 (2.5) | 0 | 3 (7.3) | 3 (3.7) |
| Adenovirus infection | 0 | 1 (2.5) | 1 (2.4) | 2 (2.5) |
| Aspartate aminotransferase increased | 0 | 1 (2.5) | 1 (2.4) | 2 (2.5) |
| COVID-19 | 2 (5.0) | 2 (5.0) | 0 | 2 (2.5) |
| Liver function test increased | 0 | 1 (2.5) | 1 (2.4) | 2 (2.5) |
| Oral herpes | 0 | 0 | 2 (4.9) | 2 (2.5) |
| Pharyngitis | 0 | 1 (2.5) | 1 (2.4) | 2 (2.5) |
| Skin abrasion | 1 (2.5) | 1 (2.5) | 1 (2.4) | 2 (2.5) |
| Acne | 0 | 1 (2.5) | 0 | 1 (1.2) |
| Alanine aminotransferase increased | 0 | 1 (2.5) | 0 | 1 (1.2) |
| Application site burn | 0 | 1 (2.5) | 0 | 1 (1.2) |
| Application site eczema | 0 | 1 (2.5) | 0 | 1 (1.2) |
| Application site erosion | 1 (2.5) | 1 (2.5) | 0 | 1 (1.2) |
| Application site folliculitis | 0 | 1 (2.5) | 0 | 1 (1.2) |
| Application site pain | 0 | 0 | 1 (2.4) | 1 (1.2) |
| Asthma | 0 | 1 (2.5) | 0 | 1 (1.2) |
| Corneal disorder | 0 | 1 (2.5) | 0 | 1 (1.2) |
| Contact dermatitis | 3 (7.5) | 0 | 1 (2.4) | 1 (1.2) |
| Hordeolum | 0 | 0 | 1 (2.4) | 1 (1.2) |
| Hyperlipidaemia | 0 | 1 (2.5) | 0 | 1 (1.2) |
| Injury | 0 | 0 | 1 (2.4) | 1 (1.2) |
| Pain in extremity | 0 | 0 | 1 (2.4) | 1 (1.2) |
| Protein urine present | 0 | 0 | 1 (2.4) | 1 (1.2) |
| Rhinitis | 2 (5.0) | 1 (2.5) | 0 | 1 (1.2) |
| Streptococcal infection | 0 | 0 | 1 (2.4) | 1 (1.2) |
| Urinary occult blood positive | 0 | 1 (2.5) | 0 | 1 (1.2) |
| Vomiting | 0 | 1 (2.5) | 0 | 1 (1.2) |
| Vulvovaginal pruritus | 0 | 1 (2.5) | 0 | 1 (1.2) |
| Xeroderma | 0 | 0 | 1 (2.4) | 1 (1.2) |
| Burns second degree | 1 (2.5) | 0 | 0 | 0 |
| Conjunctivitis | 1 (2.5) | 0 | 0 | 0 |

| AE terms | Vehicle (n = 40) | Tapinarof | | |
|---|---|---|---|---|
| | | 0.5% (n = 40) | 1% (n = 41) | Total (n = 81) |
| Contusion | 1 (2.5) | 0 | 0 | 0 |
| AD | 6 (15.0) | 0 | 0 | 0 |
| Epiphysitis | 1 (2.5) | 0 | 0 | 0 |
| Impetigo | 1 (2.5) | 0 | 0 | 0 |
| Ligament sprain | 1 (2.5) | 0 | 0 | 0 |
| Skin infection | 1 (2.5) | 0 | 0 | 0 |

Abbreviations: AD, atopic dermatitis; AE, adverse event; COVID-19, coronavirus disease 2019.

Data are presented as number of patients (%). The AE terms reported by the investigator were coded using Medical Dictionary for Regulatory Activities Terminology V.24.0.

**Table S5** Adverse events by age category occurring in ≥ 2 patients in either of the treatment groups.

| Age category | Vehicle (n = 40) | | Tapinarof 0.5% (n = 40) | | Tapinarof 1% (n = 41) | | Tapinarof Total (n = 81) | |
|---|---|---|---|---|---|---|---|---|
| | 2-6 years (n = 15) | 7-11 years (n = 25) | 2-6 years (n = 17) | 7-11 years (n = 23) | 2-6 years (n = 20) | 7-11 years (n = 21) | 2-6 years (n = 37) | 7-11 years (n = 44) |
| Any AEs | 12 (80.0) | 11 (44.0) | 10 (58.8) | 12 (52.2) | 11 (55.0) | 13 (61.9) | 21 (56.8) | 25 (56.8) |
| Gastroenteritis | 2 (13.3) | 0 | 1 (5.9) | 1 (4.3) | 3 (15.0) | 2 (9.5) | 4 (10.8) | 3 (6.8) |
| Application site irritation | 1 (6.7) | 4 (16.0) | 1 (5.9) | 1 (4.3) | 0 | 3 (14.3) | 1 (2.7) | 4 (9.1) |
| Nasopharyngitis | 1 (6.7) | 0 | 1 (5.9) | 0 | 4 (20.0) | 0 | 5 (13.5) | 0 |
| Headache | 0 | 0 | 0 | 1 (4.3) | 1 (5.0) | 2 (9.5) | 1 (2.7) | 3 (6.8) |
| Pyrexia | 0 | 1 (4.0) | 0 | 0 | 1 (5.0) | 2 (9.5) | 1 (2.7) | 2 (4.5) |
| Upper respiratory tract inflammation | 1 (6.7) | 0 | 0 | 0 | 1 (5.0) | 2 (9.5) | 1 (2.7) | 2 (4.5) |
| COVID-19 | 2 (13.3) | 0 | 1 (5.9) | 1 (4.3) | 0 | 0 | 1 (2.7) | 1 (2.3) |
| Oral herpes | 0 | 0 | 0 | 0 | 1 (5.0) | 1 (4.8) | 1 (2.7) | 1 (2.3) |
| Contact dermatitis | 1 (6.7) | 2 (8.0) | 0 | 0 | 0 | 1 (4.8) | 0 | 1 (2.3) |
| Rhinitis | 1 (6.7) | 1 (4.0) | 1 (5.9) | 0 | 0 | 0 | 1 (2.7) | 0 |
| AD | 3 (20.0) | 3 (12.0) | 0 | 0 | 0 | 0 | 0 | 0 |

Abbreviations: AD, atopic dermatitis; AE, adverse event; COVID-19, coronavirus disease 2019.

Data are presented as number of patients (%).

**Table S6** Summary of plasma concentrations of tapinarof.

| | | Week 2[a] | Week 4 | Week 8 |
|---|---|---|---|---|
| Tapinarof 0.5% | | | | |
| Number of patients | | 30 | 35 | 33 |
| Patients with a ≥ LLOQ value, n (%) | | 2 (6.7) | 10 (28.6) | 6 (18.2) |
| Mean plasma concentration (SD), pg/mL | | 5.140 (20.32) | 47.11 (99.44) | 349.4 (1913) |
| Maximum plasma concentration, pg/mL | | 98.1 | 400 | 11000 |
| Tapinarof 1% | | | | |
| Number of patients | | 31 | 37 | 34 |
| Patients with a ≥ LLOQ value, n (%) | | 8 (25.8) | 10 (27.0) | 10 (29.4) |
| Mean plasma concentration (SD), pg/mL | | 35.96 (76.82) | 27.80 (50.73) | 50.37 (105.3) |
| Maximum plasma concentration, pg/mL | | 276 | 178 | 490 |
| **By age category** | | | | |
| Tapinarof 0.5% | | | | |
| 2-6 years | Number of patients | 9 | 15 | 15 |
| | Patients with a ≥ LLOQ value, n (%) | 0 | 4 (26.7) | 3 (20.0) |
| 7-11 years | Number of patients | 21 | 20 | 18 |
| | Patients with a ≥ LLOQ value, n (%) | 2 (9.5) | 6 (30.0) | 3 (16.7) |
| Tapinarof 1% | | | | |
| 2-6 years | Number of patients | 11 | 18 | 16 |
| | Patients with a ≥ LLOQ value, n (%) | 2 (18.2) | 5 (27.8) | 4 (25.0) |
| 7-11 years | Number of patients | 20 | 19 | 18 |
| | Patients with a ≥ LLOQ value, n (%) | 6 (30.0) | 5 (26.3) | 6 (33.3) |

Abbreviations: LLOQ, lower limit of quantification; SD, standard deviation.

For the analysis of plasma concentration, < LLOQ values (< 50 pg/mL) were treated as 0.

[a]For patients aged ≤ 6 years, blood collections at week 2 were optional and performed when possible.

## Supplementary Figures

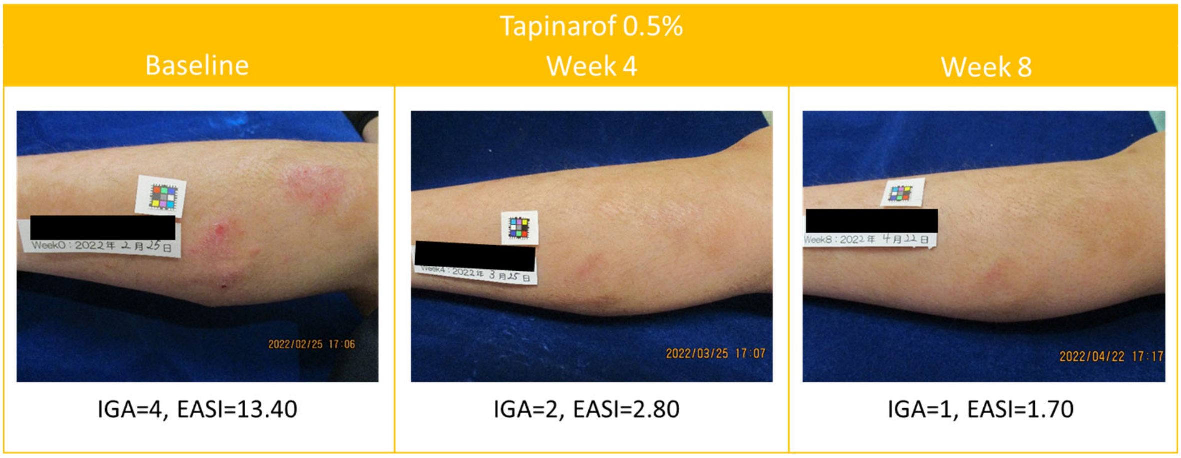


**Patient 1 (Tapinarof 0.5%), lower leg**

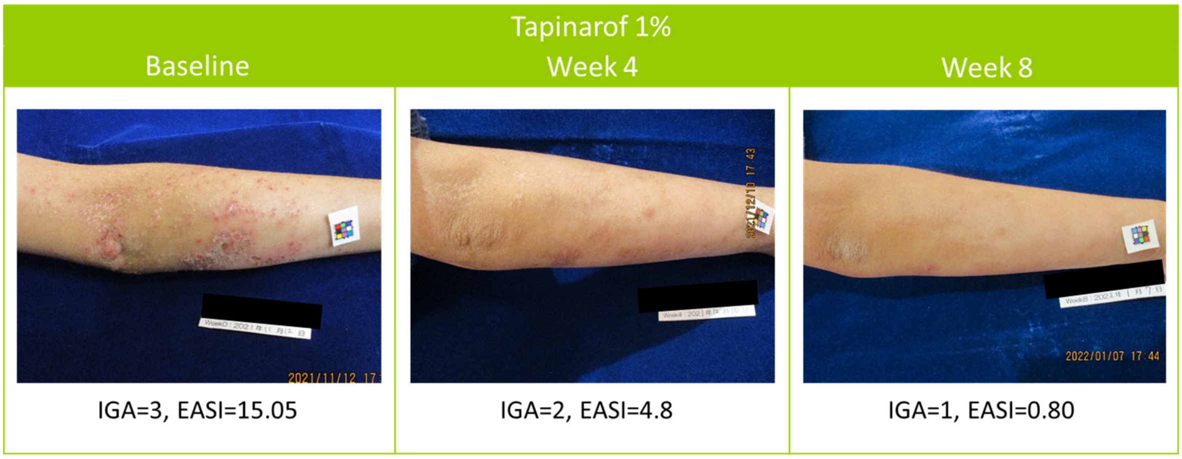


**Patient 2 (Tapinarof 1%), forearm**

**Figure S1** Representative clinical images of patients receiving tapinarof who achieved IGA treatment success. EASI, Eczema Area and Severity Index; IGA, Investigator's Global Assessment. IGA treatment success was defined as an IGA score of 0 or 1 with ≥ 2-grade improvement from baseline.

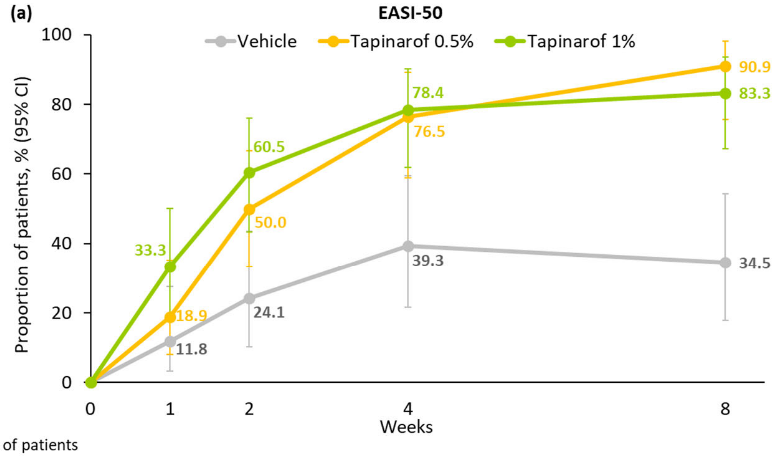


Number of patients

| | 0 | 1 | 2 | 4 | 8 |
|---|---|---|---|---|---|
| Vehicle | 40 | 34 | 29 | 28 | 29 |
| Tapinarof 0.5% | 40 | 37 | 38 | 34 | 33 |
| Tapinarof 1% | 41 | 39 | 38 | 37 | 36 |

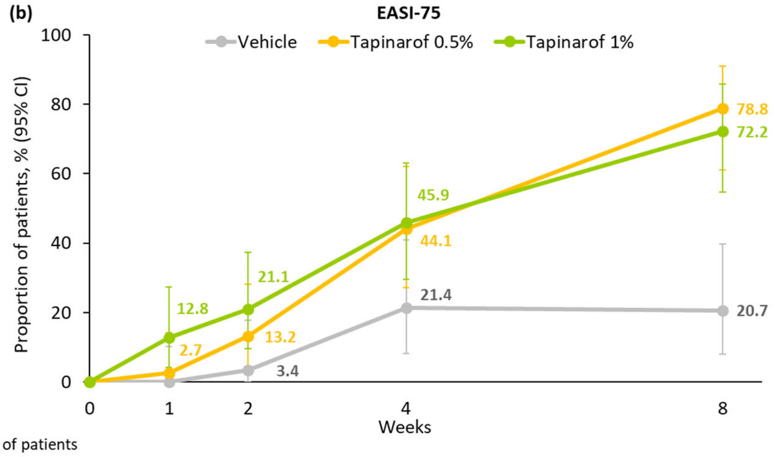


Number of patients

| | 0 | 1 | 2 | 4 | 8 |
|---|---|---|---|---|---|
| Vehicle | 40 | 34 | 29 | 28 | 29 |
| Tapinarof 0.5% | 40 | 37 | 38 | 34 | 33 |
| Tapinarof 1% | 41 | 39 | 38 | 37 | 36 |

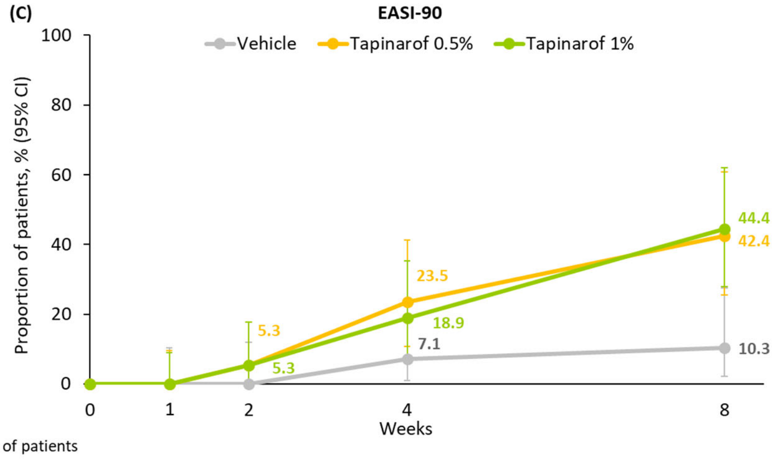


Number of patients

| | 0 | 1 | 2 | 4 | 8 |
|---|---|---|---|---|---|
| Vehicle | 40 | 34 | 29 | 28 | 29 |
| Tapinarof 0.5% | 40 | 37 | 38 | 34 | 33 |
| Tapinarof 1% | 41 | 39 | 38 | 37 | 36 |

(d)

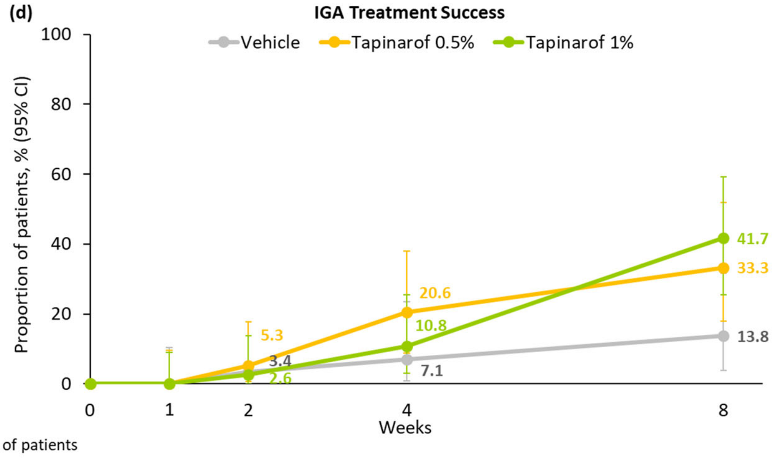


Number of patients

| | | | | | |
|---|---|---|---|---|---|
| Vehicle | 40 | 34 | 29 | 28 | 29 |
| Tapinarof 0.5% | 40 | 37 | 38 | 34 | 33 |
| Tapinarof 1% | 41 | 39 | 38 | 37 | 36 |

(e)

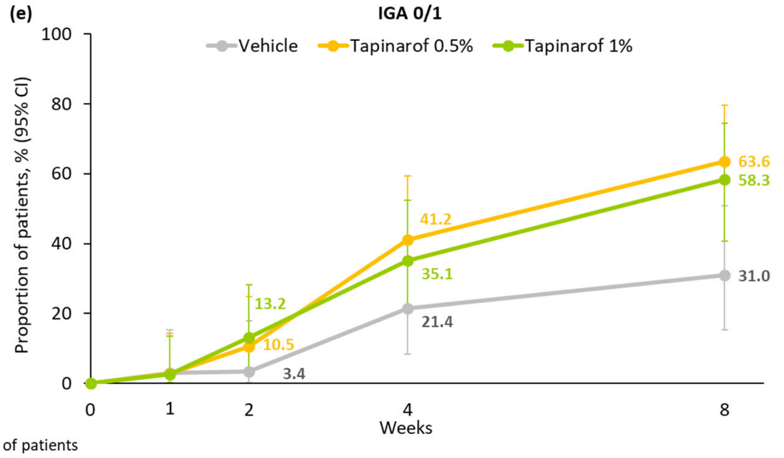


Number of patients

| | | | | | |
|---|---|---|---|---|---|
| Vehicle | 40 | 34 | 29 | 28 | 29 |
| Tapinarof 0.5% | 40 | 37 | 38 | 34 | 33 |
| Tapinarof 1% | 41 | 39 | 38 | 37 | 36 |

(f)

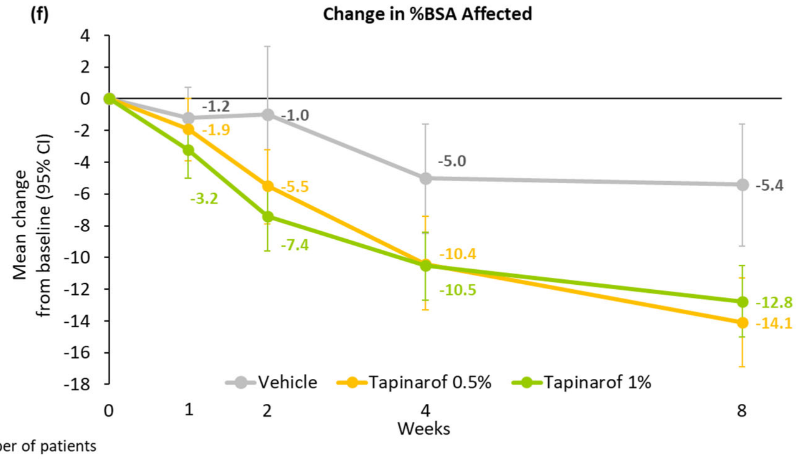


Number of patients

| | | | | | |
|---|---|---|---|---|---|
| Vehicle | 40 | 34 | 29 | 28 | 29 |
| Tapinarof 0.5% | 40 | 37 | 38 | 34 | 33 |
| Tapinarof 1% | 41 | 39 | 38 | 37 | 36 |

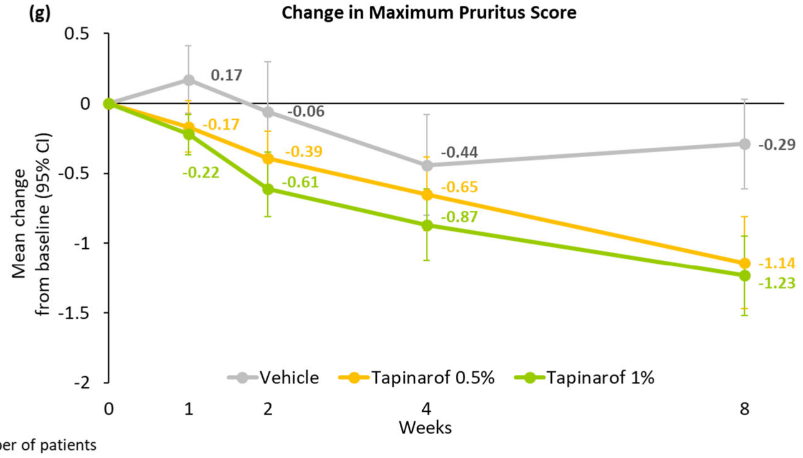


| Number of patients | | | | | |
|---|---|---|---|---|---|
| Vehicle | 40 | 35 | 29 | 28 | 29 |
| Tapinarof 0.5% | 40 | 38 | 38 | 34 | 33 |
| Tapinarof 1% | 41 | 39 | 38 | 37 | 36 |

**Figure S2** Efficacy endpoints by trial visit. (a) EASI-50 response rate, (b) EASI-75 response rate, (c) EASI-90 response rate, (d) IGA treatment success rate, (e) Proportion of patients with an IGA score of 0 or 1, (f) change from baseline in %BSA affected, and (g) change from baseline in maximum pruritus score. BSA, body surface area; CI, confidence interval; EASI, Eczema Area and Severity Index; IGA, Investigator's Global Assessment. EASI-50, -75, and -90 were defined as ≥ 50%, ≥ 75%, and ≥ 90% improvement from baseline in EASI score, respectively. IGA treatment success was defined as an IGA score of 0 or 1 with ≥ 2-grade improvement from baseline. The maximum pruritus score on an assessment day is defined as the greater of the daytime and nighttime scores. The maximum pruritus score at each visit was defined as the mean value of daily maximum pruritus scores obtained during 7 days prior to the relevant visit. Data were analyzed on the basis of observed cases (OC) where missing data were not imputed. (a) to (e) are presented with exact 95% CIs.

**References for Supporting Information**

1. Saeki H, Ohya Y, Furuta J, Arakawa H, Ichiyama S, Katsunuma T, et al. English Version of Clinical Practice Guidelines for the Management of Atopic Dermatitis 2021. J Dermatol. 2022 Oct;49(10):e315-e375. doi: 10.1111/1346-8138.16527.
